# Systems mapping: how to improve the genetic mapping of complex traits through design principles of biological systems

**DOI:** 10.1186/1752-0509-5-84

**Published:** 2011-05-27

**Authors:** Rongling Wu, Jiguo Cao, Zhongwen Huang, Zhong Wang, Junyi Gai, Eduardo Vallejos

**Affiliations:** 1 Center for Computational Biology, National Engineering Laboratory for Tree Breeding, Key Laboratory of Genetics and Breeding in Forest Trees and Ornamental Plants, Beijing Forestry University, Beijing 100083, China; 2 Department of Statistics & Actuarial Science, Simon Fraser University, Burnaby, B.C. Canada V5A 1S6; 3 Department of Agronomy, Henan Institute of Science and Technology, Xinxiang 453003, China; 4 Center for Statistical Genetics, Pennsylvania State University, Hershey, PA 17033, USA; 5 National Center for Soybean Improvement, National Key Laboratory for Crop Genetics and Germplasm Enhancement, Soybean Research Institute, Nanjing Agricultural University, Nanjing 210095, China; 6 Department of Horticultural Sciences, University of Florida, Gainesville, FL 32611, USA

## Abstract

**Background:**

Every phenotypic trait can be viewed as a "system" in which a group of interconnected components function synergistically to yield a unified whole. Once a system's components and their interactions have been delineated according to biological principles, we can manipulate and engineer functionally relevant components to produce a desirable system phenotype.

**Results:**

We describe a conceptual framework for mapping quantitative trait loci (QTLs) that control complex traits by treating trait formation as a dynamic system. This framework, called systems mapping, incorporates a system of differential equations that quantifies how alterations of different components lead to the global change of trait development and function through genes, and provides a quantitative and testable platform for assessing the interplay between gene action and development. We applied systems mapping to analyze biomass growth data in a mapping population of soybeans and identified specific loci that are responsible for the dynamics of biomass partitioning to leaves, stem, and roots.

**Conclusions:**

We show that systems mapping implemented by design principles of biological systems is quite versatile for deciphering the genetic machineries for size-shape, structural-functional, sink-source and pleiotropic relationships underlying plant physiology and development. Systems mapping should enable geneticists to shed light on the genetic complexity of any biological system in plants and other organisms and predict its physiological and pathological states.

## Background

Predicting the phenotype from the genotype of complex organisms is one of the most important and challenging questions we face in modern biology and medicine [1]. Genetic mapping, dissecting a phenotypic trait to its underlying quantitative trait loci (QTLs) through the use of molecular markers, has proven powerful for establishing genotype-phenotype relationships and predicting phenotypes of individual organisms based on their QTL genotypes responsible for the trait [2]. The success of this prediction depends on how well we can map the underlying QTLs and characterize complex interactions of these QTLs with each other and with environmental factors. Powerful statistical models have been developed in the past two decades to detect QTLs and study their biological function in a diverse array of phenotypic traits [3-9]. Worldwide, a substantial effort has been made resulting in the collection of a large amount of data aimed at the identification of QTLs [10-15]. Unfortunately, despite hundreds of thousands of QTLs detected in a diversity of organisms, only a few of them have been isolated by positional cloning (see [16-18]), leaving it unsolved how to construct a genotype-phenotype relationship map using genetic mapping.

The most likely reason for this result may arise from a possibility that the QTLs detected by stringent statistical tests are not biologically relevant. Existing strategies for QTL mapping were built on testing for a direct association between genotype and end-point phenotype. Although such strategies are simple and have been widely accepted, they neglect the biological processes involved in trait development [14]. To attempt to fill this gap, a statistical model, called functional mapping [19-21], has been developed to study the interplay between genetics and the developmental process of a phenotypic trait by integrating mathematical models and computational algorithms. If a trait is understood as a "system" that is composed of many underlying biological components [22-24], we should be in a better position to comprehend the process and behavior of trait formation based on interactive relationships among different components. Through mapping and using those QTLs that govern design principles of a biological system, a new trait that is able to maximize resource-use efficiency can be generated and engineered.

As one important strategy for plants to respond to variation in the availability of resources in their environment, biomass allocation has been extensively used to study the relationship between structure and function in modern ecology [25-28]. The concept of biomass allocation has now been increasingly integrated with plant management and breeding, aimed to direct a maximum amount of biomass to the target of harvest (leaves, stem, roots, or fruits) [29-31]. If the whole-plant biomass is considered as a target trait, we need to understand how different organs of a plant coordinate and interact to optimize the capture of nutrients, light, water, and carbon dioxide in a manner that maximizes plant growth rate through a specific developmental program because plant biomass growth is not simply the addition of biomass to various organs. Many theories and models have been proposed to predict the pattern of biomass partitioning in a response to changing environment. Chen and Reynolds [27] used coordination theory to model the dynamic allocation of carbon to different organs during growth in relation to carbon and water/nitrogen supply by a group of differential equations. Compared to the conventional optimization model in the context of maximizing the relative growth rate of a plant, the coordination model does not require an unrealistic capacity the plant possesses for knowing beforehand the environmental conditions it will experience during the growth period. Here, we integrate the coordination and optimization model to study the pattern of biomass partitioning by incorporating the allometric scaling theory into a system of differential equations.

In a series of allometric studies by West et al. [32-34], a power relationship that universally exists between parts and the whole can be explained by two fundamental design principles in biophysics and biochemistry; i.e., all organisms tend to maximize their metabolic capacity by increasing surface areas for energy and material production as well as internal efficiency through reducing distances and the time to transport water, nutrients, and carbon. The integration of this optimization theory expressed in terms of allometric scaling with the coordination theory leads to a tripled group of ordinary differential equations (ODEs) to specify the coordination of the leaf, stem, and root biomass for a plant:(1)

where *M_L_*, *M_S_*, and *M_R_*are the biomasses of the leaves (*L*), the stem (*S*), and the roots (*R*), respectively, with whole-plant biomass *W *= *M_L_*+ *M_S_*+ *M_R_*; *α *and *β *are the constant and exponent power of an organ biomass scaling as whole-plant biomass [32,33]; and *γ *is the rate of eliminating ageing leaves and roots. The complex interactions between different parts of a plant that underlie design principles of plant biomass growth can be modeled and studied by estimating and testing the ODE parameters (*α_L_*, *β_L_*, *λ_L_*, *α_S_*, *β_S_*, *α_R_*, *β_R_*, *λ_R_*). For example, plants are equipped with a capacity to optimize their fitness under low nutrient availability by shifting the partitioning of carbohydrates to processes associated with nutrient uptake at a cost of carbon acquisition [29]. These parameters can be used to quantify and predict such regulation between different plant parts in response to environmental and developmental changes.

In this article, we put forward a conceptual framework to incorporate the design principles of trait formation and development into a statistical framework for QTL mapping. Complementary to our previous functional mapping [19-21], we name this new mapping framework "systems mapping" in light of its systems dissection and modeling of phenotypic formation. A group of ODEs like (1) or other types of differential equations is used to quantify the phenotypic system. Much work in solving ODEs has focused on the simulation and analysis of the behavior of state variables for a dynamic system, but the estimation of ODE parameters that define the system based on the measurement of state variables at multiple time points is relatively a new area. Yet, in the recent years, many statisticians have made great attempts to develop statistical approaches for estimating ODE parameters by modeling the structure of measurement errors [35-42]. We implemented Ramsay et al.'s [41] penalized spline method for estimating constant dynamic parameters in our genetic mapping. The problem for systems mapping with ODE models is different from those considered in current literature. First, systems mapping is constructed within a mixture-based framework because QTL genotypes that define the DE models are missing. Second, systems mapping incorporates genotypic data which are categorical or binary. These two characteristics determine the high complexity of our statistical model and computational algorithm used for systems mapping.

## Results

### QTL detection

**Figure 1:**
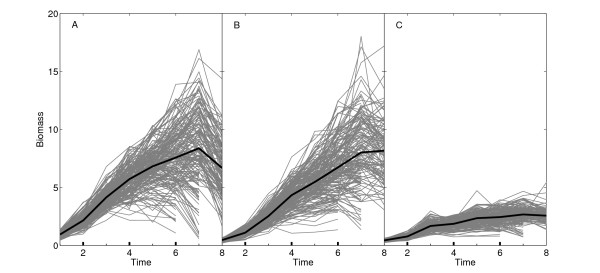
**Growth trajectories of leaf (A), stem (B) and root biomass (C) measured at multiple time points in a growing season of soybeans**. Each grey line presents the growth trajectory of one of 184 RILs, whereas black lines are the mean growth trajectories of all RILs fitted using a system of ODEs (1).

We develop a new model for QTL mapping by treating trait formation as a dynamic system and further incorporating the design principles of the biological system into a statistical mapping framework (see **Methods**). The new model, named systems mapping in light of its systems feature of phenotypic description, was used to map QTLs for biomass partitioning in a mapping population of soybeans composed of 184 recombinant inbred lines (RIL) derived from two cultivars, Kefeng No. 1 and Nannong 1138-2. For an RIL population, there are two homozygous genotypes, one composed of the Kefeng No. 1 alleles and the other composed of the Nannong 1138-2 alleles. Figure 1 illustrates the growth trajectories of leaf, stem and root biomass for individual RILs. By using the system of ODEs (1) to fit growth trajectories of leaf, stem and root biomass over time, we obtained a mean curve for each trait. It can be seen that growth trajectories can be well modeled by three interconnecting ODEs (1) derived from coordination theory [27] and allometric scaling [32-34]. The model-fitted curves of leaf (Figure 1A) and root biomass trajectories (Figure 1C) delineate reasonably the decay of leaf and root biomass at a late stage of development due to senescence. As expected, stem biomass growth does not experience such a decay (Figure 1B) although growth at the late stage tends to be stationary.

**Figure 2:**
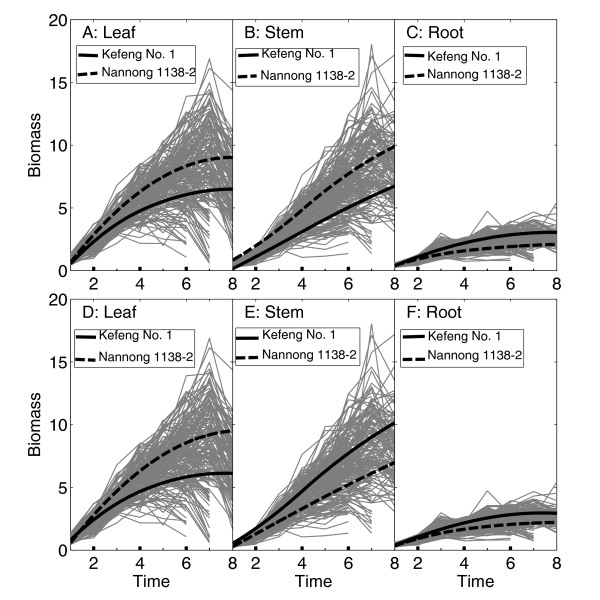
**Growth trajectories of leaf (A, D), stem (B, E) and root biomass (C, F) for two different genotypes (presented by solid and broken black curves) at a QTL detected on linkage group 12 (upper panel) and 24 (lower panel), respectively**. Two genotypes at a QTL are the homozygote for the alleles inherited from Kefeng No.1 (solid) and the homozygote for the allele from Nannong 1138-2 (broken). Curves in grey are growth trajectories of 184 RILs.

By scanning the genetic linkage map composed of 950 molecular markers located in 25 linkage groups, we detected two significant QTLs, one named as *biomass1 *that resides between markers GMKF167a and GMKF167b and the second as *biomass2 *that resides between markers sat_-_274 and BE801128 (Additional file 1, Figure S1). Using the maximum likelihood estimates of the curve parameters in ODE (1) whose standard errors were obtained by the parameter bootstrap [43] (Table 1), we drew the growth trajectories of leaf, stem and root biomass for two different genotypes at each QTL (Figure 2). The genetic effects of the QTLs displayed different temporal patterns for three organs. The QTLs were expressed more rapidly with time for the stem than for the leaves and roots. At *biomass1*, the alleles from parent Nannong 1138-2 increase leaf and stem biomass growth (Figure 2A and 2B), whereas the alleles from parent Kefeng No. 1 increase root biomass growth (Figure 2C). This could be interpreted as the Nannong 1138-2 allele favoring carbon allocation to the shoots at the expense of the roots but the Kefeng No1 allele favoring carbon allocation to the roots at the expense of the shoots. Likewise, the *biomass2 *alleles from Nannong 1138-2 favor carbon allocation to the leaves (Figure 2D) and those from Kefeng No. 1 favor carbon allocation to the roots, but the alleles at this QTL inherited from parent Kefeng No. 1 favor carbon allocation to the stem, which is different from the behavior of QTL *biomass1*. Note that leaf and root biomass growth tend to decay at the late stage for almost all RILs. But the genotypes at the QTLs detected do not reflect this trend (Figure 2), although they display much reduced rates of growth at the late stage. We explained this to be due to some other QTLs that have not been detected with the current linkage map.

**Table 1:** The maximum likelihood point estimates (PEs) of ODE parameters and standard errors (SEs) of the estimates for the QTLs detected.

QTL	Model	Genotype	Estimate	*α_L_*	*β_L_*	*γ_L_*	*α_S_*	*β_S_*	*α_R_*	*β_R_*	*γ_R_*
1	M1	*QQ*	PE	2.09	0.16	0.43	0.93	0.07	1.52	0.66	2.91
			SE	0.02	1e-3	3e-3	0.01	2e-4	0.01	6e-3	0.02
		
		*qq*	PE	2.53	0.11	0.36	0.92	0.04	1.57	0.54	3.90
			SE	0.01	4e-4	2e-3	0.01	1e-4	0.01	5e-3	0.01
	
	M0		PE	2.30	0.13	0.39	0.92	0.05	1.56	0.60	3.37
			
			SE	0.07	2e-3	0.01	0.02	4e-4	0.05	0.02	0.08

2	M1	*QQ*	PE	1.89	0.14	0.44	1.04	0.07	1.11	0.56	1.85
			SE	0.04	1e-3	0.01	0.01	1e-4	0.01	5e-3	0.01
		
		*qq*	PE	2.55	0.10	0.31	0.98	0.04	1.11	0.51	2.18
			SE	0.04	1e-3	0.01	0.01	4e-4	0.01	5e-3	0.02
	
	M0		PE	2.25	0.12	0.37	1.03	0.05	1.12	0.55	2.06
			
			SE	0.08	3e-3	0.02	0.02	6e-4	0.02	0.01	0.05

Model M1 assumes two different genotypes at a QTL (under the H_1_), whereas Model M0 assumes a single genotype (under the H_0_).

Note: QTL 1 is one between markers GMKF167a and GMKF167b on linkage group 12. QTL 2 is one between markers sat_-_274 and BE801128 on linkage group 24. The alleles of genotype *QQ *are derived from Kefeng No. 1 and those of genotype *qq *derived from Nannong 1138-2.

**Figure 3:**
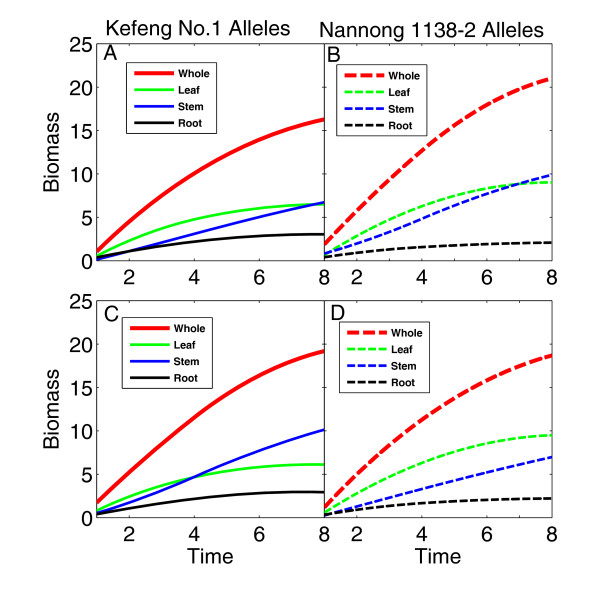
**Growth trajectories of whole-plant (red), leaf (green), stem (blue) and root biomass (black) for two different genotypes at a QTL detected on linkage group 12 (upper panel) and 24 (lower panel)**. Two genotypes at a QTL are the homozygote for the alleles inherited from Kefeng No.1 (A, C) and the homozygote for alleles inherited from Nannong 1138-2 (B, D).

The functional relationships among leaf, stem and root biomass were determined by the QTLs detected (Figure 3). For *biomass1*, two genotypes are not only different in whole-plant biomass trajectory, but also display pronounced discrepancies in biomass growth trajectories of individual organs (Figure 3A and 3B). This means that this QTL affects the dynamics of both plant size and biomass partitioning. The genotype composed of the Kefeng No. 1 alleles has a smaller slope of biomass growth, leading to smaller whole-plant biomass at late stages of development, than that composed of the Nannong 1138-2 alleles, but the former has larger root biomass over the entire period of growth at the expense of the shoots than the latter. For *biomass2*, two genotypes are similar in total plant size during growth, but they have a marked distinction in biomass partitioning (Figure 3C and 3D). It appears that this QTL affects plant growth trajectories through altering biomass partitioning rather than total amount of biomass. At this QTL, the genotype with the Kefeng No. 1 alleles has a dominant main stem and heavy roots, whereas the genotype with the Nannong 1138-2 alleles carries dense leaves.

**Figure 4:**
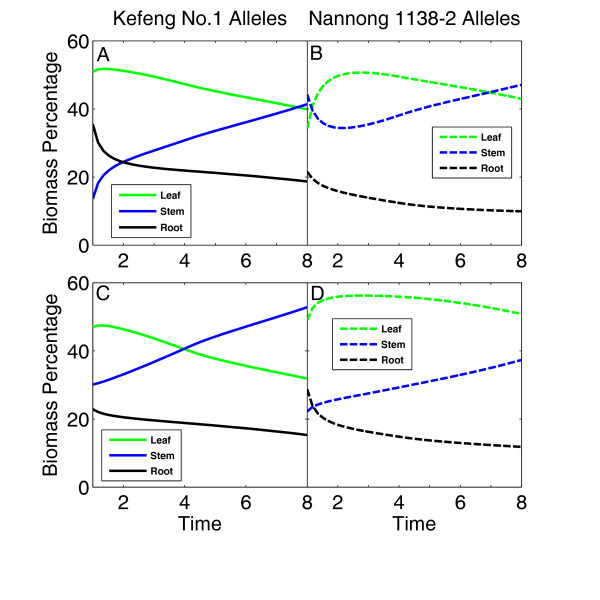
**Time-dependent percentages of leaf (green), stem (blue) and root biomass (thin black) for two different genotypes at a QTL detected on linkage group 12 (upper panel) and 24 (lower panel)**. Two genotypes at a QTL are the homozygote for the alleles inherited from Kefeng No.1 (A, D) and the homozygote for alleles from Nannong 1138-2 (B, C).

Figure 4 shows the dynamic pattern of biomass partitioning to different organs. In general, the stem receives increasing allocation with time, whereas the partitioning to the leaves and roots decreases with time. Both QTLs detected, *biomass1 *and *biomass2*, control the degree of such time-dependent increase or decrease. For example, at *biomass1*, the Kefeng No. 1 genotype always exhibits a larger degree of increasing biomass partitioning to the stem but a larger degree of decreasing biomass partitioning to the leaves and roots than the Nannong 1138-2 genotype (Figure 4A vs. 4B). QTL *biomass2 *has a similar pattern of biomass partitioning for the stem and leaves, although it displays a stronger effect than does QTL *biomass1*. At QTL *biomass2*, there is a larger degree of decreasing biomass partitioning to the roots for the Nannong 1138-2 genotype than the Kefeng No. 1 genotype (Figure 4C vs. 4D).

### Simulation

By analyzing a real data set for soybean mapping, systems mapping produces the identification of two significant QTLs that control the dynamic formation of whole-plant biomass through developmental regulation of different organs, stem, leaves, and roots. To validate the new model, we performed simulation studies by mimicking the effects of QTL *biomass2 *detected from the example of QTL mapping in soybeans. The simulated mapping population contains the same genotype data for 184 RILs. The phenotypic values of three traits, the stem, leaf and root biomass, assumed to obey the system of ODE (1), were simulated at six different time points by summing time-dependent genotypic values at *biomass2 *calculated with curve parameters in Table 1 and residual errors. Specifically, the phenotypic values of the *k*th trait were simulated by adding white noise with variances  to the ODE curves for the *j*th QTL genotype with the probability *w*_*j*|*i*_, i.e., the conditional probability of the *i*th RIL that carries the *j*th QTL genotype, given the two markers genotypes of this RIL. The values of noise variance  were set as the estimates from the real data, which are ,  and  for the leaf, stem, and root biomass, respectively. Meanwhile, by assuming a modest heritability (0.05) for each trait at a middle stage of growth, we re-scaled  values which were used to simulate a new data set.

Systems mapping, implemented with the parameter cascading method, estimates QTL genotype-specific curve parameters in the ODE (1) from the simulated data. The simulation was repeated 100 times to calculate the means, biases, standard deviations, and root mean square errors, with results tabulated in Table 2. It was found that the model can provide reasonably accurate and precise estimates of QTL genotype-specific ODE parameters with a modest sample size (*n *= 184). The biases of the estimates are negligible, compared with the scale of the standard deviations. Given that this simulated data is a mimicry of the real soybean data, the results suggest that the experimental design used for soybean mapping is scientifically sound and can provide convincing QTL detection. This actually is not surprising because phenotyping has low measurement errors.

**Table 2:** Means of maximum likelihood estimates of curve parameters from the ODE system (1) and their biases, standard deviations (STD) and square root mean square errors (RMSE) from 100 simulation replicates.

Genotype	Estimate	*α_L_*	*β_L_*	*γ_L_*	*α_S_*	*β_S_*	*α_R_*	*β_R_*	*γ_R_*
*QQ*	TRUE	2.55	0.10	0.31	0.98	0.04	1.11	0.51	2.18
	MEAN	2.55	0.10	0.31	0.98	0.04	1.11	0.51	2.18
	BIAS*10^3^	2.10	-0.07	0.72	0.27	0.01	3.49	-0.88	-2.89
	STD*10^2^	3.75	0.08	0.60	0.94	0.01	1.29	0.49	1.11
	RMSE*10^2^	3.75	0.08	0.61	0.94	0.01	1.34	0.50	1.15

*qq*	TRUE	1.89	0.14	0.44	1.04	0.07	1.11	0.56	1.85
	MEAN	1.89	0.14	0.44	1.04	0.07	1.11	0.56	1.85
	BIAS*10^3^	-4.23	0.11	0.55	-0.61	-0.05	1.62	0.92	-2.02
	STD*10^2^	3.60	0.14	0.92	0.99	0.04	1.38	0.45	1.66
	RMSE*10^2^	3.62	0.14	0.92	0.99	0.04	1.39	0.46	1.67

In analyzing a simulated data set for the traits assumed to have a modest heritability (0.05), the estimates of the ODE parameters are reasonably accurate and precise, indicating the power of systems mapping to detect small QTLs involved in trait formation. We performed an additional simulation to investigate the power and false positive rates of the model by changing levels of noises. In general, the power of the model is high, reaching 0.80 even when the heritability of growth curves is low (0.05). Basically, a QTL can be fully detected when the heritability is 0.10 or larger. In any case, the false positive rates are not beyond 0.10, mostly being less than 0.05.

## Discussion

Mapping the genetic architecture of complex traits has been a subject of interest in both theoretical and empirical aspects of modern biology [3-15]. Original approaches for genetic mapping are based on single-point variation in a phenotypic trait, neglecting the dynamic change of the trait during development. To capture the dynamic pattern of genetic control, a new statistical model called functional mapping has been developed by incorporating the mathematical aspects of trait development [19-21]. Despite significant improvement over conventional static mapping, functional mapping has still a major limitation in characterizing developmental pathways that cause a final phenotype and unraveling the underlying genetic mechanisms for trait formation and progression.

In this article, we have for the first time put forward a new approach-systems mapping by treating a phenotypic trait as a dynamic system and incorporating the design principles of a biological system into a statistical framework for genetic mapping. Various components that constitute the system through developmental regulation are studied and connected by a system of biologically meaningful differential equations (DE). Thus, the genetic mapping of a complex phenotype become an issue of testing and estimating genotype-specific curve parameters at specific QTLs that define the emerging properties and dynamic behavior of the DE system. Systems mapping identifies QTLs that control developmental interactions of traits, the temporal pattern of QTLs expression during development, as well as the genetic determinants that control developmental switches (on/off).

Systems mapping was applied to map QTLs for dynamic trajectories of biomass from different organs, the stem, leaves, and roots, that interact and coordinate to determine whole-plant biomass growth, in an experimental cross of soybeans between Kefeng No. 1 and Nannong 1138-2. In general, the stem receives a proportionally larger amount of biomass with development, accompanying a proportional decrease of biomass to the leaves and roots. Specific QTLs, *biomass1 *and *biomass2*, that control this allometric change with development have been detected from systems mapping. The alleles at the two QTLs inherited from Kefeng No. 1 tend to amplify this contrast in development-dependent biomass partitioning, as compared to those from Nannong 1138-2. One of the two QTLs, *biomass2*, was found in a similar genomic region identified by traditional functional mapping [44]. This consistency does not only simply verify our systems mapping, but also gains new insight into biological functions of the detected QTLs. For example, the two QTLs detected, *biomass1 *and *biomass2*, trigger genetic effects on the interactions and coordination of different organs which cause the dynamic variation of biomass growth.

Through various tests for ODE parameters individually or in a combination, our systems mapping can reveal the genetic control mechanisms for several mechanistically meaningful relationships. They are (1) *size-shape relationship *- is a big plant due to a big stem with sparse leaves or a small stem with dense leaves? (2) *structural-functional relationship *- in a specific environment does a plant tend to allocate more carbon to its leaves for CO_2 _uptake or roots for water and nutrient uptake? (3) *cause-effect relationship *- are more roots due to more leaves or do more leaves produce more roots? and (4) *pleiotropic relationship *- different traits with a similar function tend to integrate into modularity [45]. How do the same QTLs pleiotropically control this modularity? A better understanding of these relationships helps to gain more insights into the mechanistic response of plant size and shape to developmental and environmental signals and, also, provide guidance to select an ideotype of crop cultivars with optimal shape and structure suited to a particular environment [46].

The model described in this article is a simple framework for systems mapping. It can be used as a start point to expand the concept of systems mapping to tackle more complicated biological problems. A phenotype can be dissected to any number of components at any level of organization, molecule, cell, tissue, or whole organism, depending on the interest of researchers and data availability. With more knowledge about phenotype formation and development, more components can be involved in a system that is specified by high-dimension differential equations. Sophisticated mathematical techniques are needed to obtain stable solutions of these equations. In addition, by integrating it with genome-wide association studies, systems mapping will not only provide a clear view of how different components interact and coordinate to form a phenotype, but also will be capable of illustrating a comprehensive picture of the genetic architecture of complex phenotypes. There is also a good reason to integrate systems mapping with network biology to explore how "omics" information contribute to the regulatory mechanisms of phenotype formation [47]. In any case, systems mapping will open a new avenue for understanding the genetic architecture of complex phenotypes from a perspective of mechanistic pathways inside their formation.

## Conclusions

The past two decades have seen a phenomenal increase in the number of tools for the genetic mapping of complex traits. Although genetic mapping continues to be an interesting area in genetic research owing to the success of molecular and sequencing technologies in generating a flood of data, a conceptual breakthrough in this area remains elusive. In this article, we present a bottom-top model for mapping and studying the genetic architecture of complex traits. Different from existing mapping models, we use a systems approach to identify specific genes or quantitative trait loci that govern the developmental interactions of various components comprising the phenotype. The map of developmental interactions among different components is constructed by a system of differential equations. Thus, by estimating and testing mathematical parameters that specify the system, we are able to predict or alter the physiological status of a phenotype based on the underlying genetic control mechanisms. We have tested and validated our model by analyzing a real data set for genetic mapping of biomass growth in soybeans. The detection of QTLs by the new model provides biologically meaningful interpretations of QTL effects on trait formation and dynamics. The new model can be readily used to study the genetic basis of phenotypes in any other organism.

## Methods

### Mapping Population

Our model derivation is based on a mapping population comprising of *n *recombinant inbred lines (RILs), initiated with two inbred lines. By continuous selfing or inbreeding, RILs after the F_7 _generation are considered homozygous because the fixation at any locus is given by *f *= 1 - 0.5^7-1 ^≈ 1. In practical terms, all plants from a single RIL are genetically identical, and can be used for replicated experiments under different environments. In addition, each RIL represents a unique combination of alleles from the parental genotypes where there are two homozygous genotypes at each marker locus, each corresponding to a parental allele. The mapping population is genotyped at molecular markers to construct a linkage map covering the entire genome. The recombination fraction between two markers is converted to the genetic distance in centiMorgan (cM) through a map function, such as the Haldane or Kosambi map function. The map constructed is used to locate QTLs that control a quantitative trait of interest.

We obtained a sample of 184 RILs derived from two cultivars, Kefeng No. 1 and Nannong 1138-2, for mapping agronomic traits. These RILs were genotyped for 950 molecular markers locating in 25 linkage groups [48,49]. The plants were grown in a simple lattice design with two replicates in a plot at Jiangpu Soybean Experiment Station, Nanjing Agricultural University, China. Ten plants in the second row of a plot were randomly selected for measuring leaf, stem and root biomass at each time in the whole growing season. After 20 days of seedling emergence, dry weights separately for the leaves, stem and roots were measured once every 5 to 10 days until most plants stopped growth. A total of 6 to 8 measurements were taken for each of the RILs studied. Great efforts were made to control measurement errors for such a large-scale field trial. Phenotyping precision was estimated to be above 95%.

Unlike a traditional mapping project, our goal is to map QTLs that control the dynamic process of how different organs, the stem, leaves, and roots, interact and coordinate to determine whole-plant biomass. The interactions and coordination of different organs for a plant are understood using design principles described by the ODE system (1).

### Likelihood

Let  denote the vector of phenotypic values for trait *k *(*k *= 1 for leaf biomass (*L*), 2 for stem biomass (*S*), and 3 for root biomass (*R*)) measured on progeny *i *at time points . Note that the number of time points measured may be progeny-specific, expressed as *m_i_*for progeny *i*. Assuming that multiple QTLs (segregating with *J *genotypes), each bracketed by two flanking markers **M**, affects these three traits, we construct a mixture model-based likelihood as(2)

where **y **= (**y**_1_, **y**_2_, **y**_3_) is a joint vector of phenotypic values for the three traits, with **z***_i_*= (**y**_1*i*_, **y**_2*i*_, **y**_3*i*_) presenting the **z**-vector for progeny *i*; *ω*_*j*|*i *_is the conditional probability of QTL genotype *j *(*j *= 1,..., *J*) given the marker genotype of progeny *i*, which can be expressed as a function of the recombination fractions between the QTL and markers [50], and *f_j_*(**z***_i_*; **Θ***_j_*, **Ψ**) is an MVN of leaf, stem and root biomass for progeny *i *which carries QTL genotype *j*, with mean vectors

specified by **Θ***_j_*, and covariance matrix specified by **Ψ**. If a system of differential equations (1) is used to jointly model QTL genotype-specific means vectors for the three traits, then we have **Θ***_j_*= (*α_Lj_*, *β_Lj_*, *λ _jL_*, *α_Sj_*, *β_Sj_*, *α_Rj_*, *β_Rj_*, *λ _Rj_*) for genotype *j*.

### Estimation

Unlike a traditional mixture model for QTL mapping, we will model the genotypic values of each QTL genotype in likelihood (2) characterized by a group of nonlinear ODEs. While an analytical solution is not available, we will implement numerical approaches to solve these ODEs. Let *μ_kj_*(*t*) denote the genotypic value of the *k*th trait at time *t *for a QTL genotype *j*. Thus, the dynamic system of the traits and their interactions, regulated by QTL genotypes, can be modeled by a system of ODE (1),(3)

where ***μ****_j_*(*t*) = (*μ*_1*j*_(*t*), ⋯, *μ*_3*j*_(*t*))*^T^*, and **Θ***_j _*is a vector of ODE parameters associated with QTL genotype *j*. For *J *possible genotypes in the mapping population, we have . The question now is how to **Θ **estimate from noisy measurements. The functional mean *μ_kj_*(*t*) may be represented as a linear combination of basis functions:(4)

where ***ϕ****_kj_*(*t*) *= *(*ϕ*_*kj*1_(*t*), ⋯, *ϕ_kjR_*(*t*))*^T^*is a vector of basis functions with *R *orders and **c***_kj_*= (*c*_*kj*1_, ⋯, *c_kjR_*)*^T ^*is a vector of basis coefficients. Define  as a length (*R *× 3 × *J*) vector of basis coefficients. The cubic B-splines are often chosen as basis functions, since any B-spline basis function is only positive over a short subinterval and zero elsewhere. This is called the *compact support *property, and is essential for efficient computation. The flexibility of the B-spline basis functions depend on the number and location of knots we choose. It is an infinite-dimension optimization problem to choose the optimal number of knots and their locations. A popular approach to avoid this dilemma is choosing a saturated number of knots and using a roughness penalty to control the smoothness of the fitted curve and avoid over-fitting [40].

We estimate the basis coefficient **c **and ODE parameter **Θ **based on a two-nested level of optimization. In the inner level of optimization, **c **is estimated by optimizing a criterion *U*(**c**|**Θ**), given any value of **Θ**. Therefore, the estimate  may be viewed as a function of **Θ**, which is denoted as . Since no analytic formula for  is available,  is an implicit function. In the outer level of optimization, **Θ **is estimated by optimizing a criterion . The parameter  is removed in the parameter space in the outer level by treating it as an implicit function of **Θ**. Although  does not have an analytic formula, the outer level of optimization only requires to calculate the derivative , which can be obtained by using the implicit function theorem. The above optimization procedure is called the parameter cascading method. Note that when the two criteria *U*(**c**|**Θ**) and  are the same, the parameter cascading method is equivalent to the profiling method.

## Authors' contributions

RW conceived of the idea of systems mapping, coordinated the whole study, and wrote the manuscript. JC derived the model, computed the real data, run simulation studies, and participated in the writing of the Methods section. ZH conducted the soybean experiment and collected phenotypic data. ZW packed the model into a computer package SysMap. JG directed the experimental design and data collection of soybeans. EV oversaw the project and highlighted the biological relevance a computational model must possess. All authors read and approved the final manuscript.

## Supplementary Material

Additional file 1 **Figure S1**. The profiles of the log-likelihood ratios (LR) between the full model (there is a QTL) and reduced model (there is no QTL) for soybean height growth trajectories throughout the soybean genome composed of 25 linkage groups. Click here for file
