# Supplementary material for: Systems mapping: how to improve the genetic mapping of complex traits through design principles of biological systems
Source: BMC Syst Biol. 2011 May 27;5:84. doi: 10.1186/1752-0509-5-84 (PMC3127792; doi:10.1186/1752-0509-5-84)
Supplement: Additional file 1 — Figure S1. The profiles of the log-likelihood ratios (LR) between the full model (there is a QTL) and reduced model (there is no QTL) for soybean height growth trajectories throughout the soybean genome composed of 25 linkage groups. [file 1752-0509-5-84-S1.PDF]

Supplementary Material

A conceptual framework for genetic dissection of complex traits through design principles of biological systems

Rongling Wu[1*], Jiguo Cao[2*], Zhongwen Huang[3], Zhong Wang[4], Junyi Gai[5] and C. Eduardo Vallejos[6]

[1] *Center for Computational Biology, National Engineering Laboratory for Tree Breeding, Key Laboratory of Genetics and Breeding in Forest Trees and Ornamental Plants, Beijing Forestry University, Beijing 100083, China*

[2] *Department of Statistics & Actuarial Science, Simon Fraser University, Burnaby, B.C. Canada V5A 1S6*

[3] *Department of Agronomy, Henan Institute of Science and Technology, Xinxiang 453003, China*

[4] *Center for Statistical Genetics, Pennsylvania State University, Hershey, PA 17033, USA*

[5] *National Center for Soybean Improvement, National Key Laboratory for Crop Genetics and Germplasm Enhancement, Soybean Research Institute, Nanjing Agricultural University, Nanjing 210095, China*

[6] *Department of Horticultural Sciences, University of Florida, Gainesville, FL 32611, USA*

Additional file, Figure S1

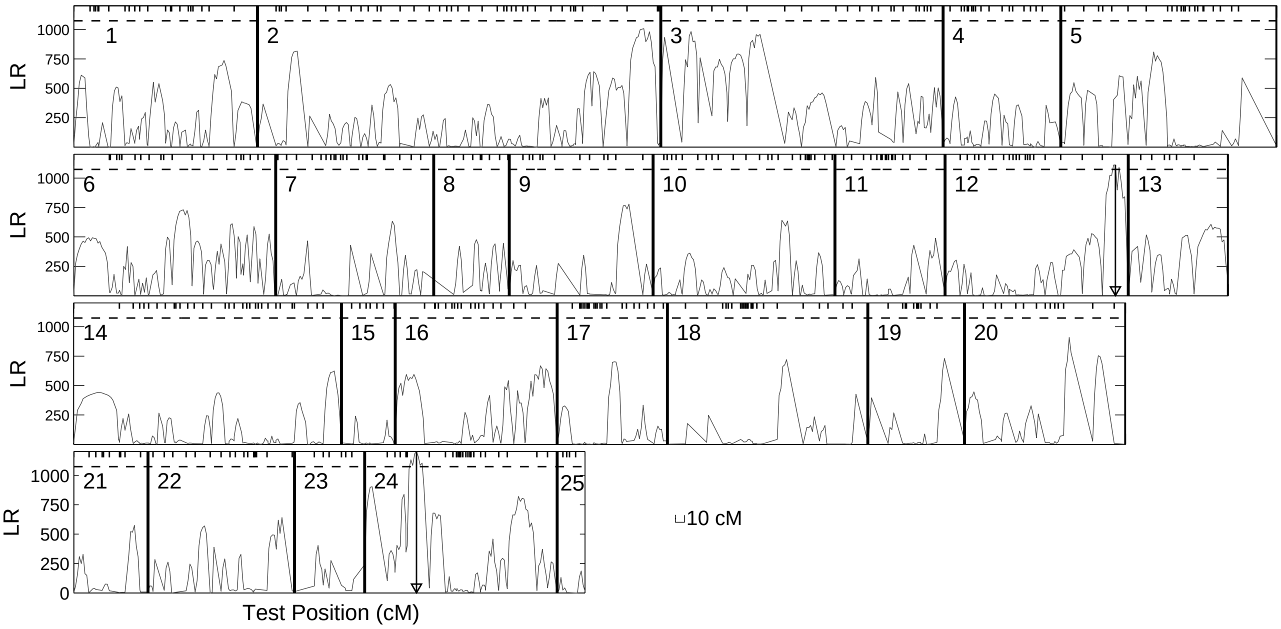

The profiles of the log-likelihood ratios (LR) between the full model (there is a QTL) and reduced model (there is no QTL) for soybean height growth trajectories throughout the soybean genome composed of 25 linkage groups. The genomic position corresponding to the peak of the curve is the maximum-likelihood estimate of the QTL location. Tick marks on the x-axis represent the positions of microsatellite markers on each chromosome (bar, 10 cM). The critical thresholds for acclaiming the genome-wide existence of a QTL are obtained from permutation tests. The 95th percentile (indicated at horizontal lines) of the distribution of the maximum LR values obtained from 200 permutation tests is used as an empirical critical value to declare genome-wide existence of a QTL at the 5% significance level.
